# Supplementary material for: Sustained-input switches for transcription factors and microRNAs are central building blocks of eukaryotic gene circuits
Source: Genome Biol. 2013 Aug 23;14(8):R85. doi: 10.1186/gb-2013-14-8-r85 (PMC4054853; doi:10.1186/gb-2013-14-8-r85)
Supplement: Additional file 5 — HTML Browsable Motif Output. Zipped folder containing all WaRSwap and FANMOD motif output, viewable in a web browser. [file gb-2013-14-8-r85-S5.ZIP › HTML_browsable_motif_output/WaRSwap_all_tair9/sigs_WSWRSLDraw-2000_all.pvals.heatmaps.html/motif_id_14_011110001_tftype_all_upstream_-2000_0.html]

```
BG_MODEL = Rnew2
MOTIF_ID = 14_011110001
TF_TYPE = all
UPSTREAM = -2000_0


PVals
FN_0.2	FN_0.4	FN_0.6	FN_0.8
dg_60.genes	0.299	0.003	0.031	0.673
dg_70.genes	0.224	0.003	0.036	0.649
dg_80.genes	0.665	0.093	0.28	0.542

ZScores
FN_0.2	FN_0.4	FN_0.6	FN_0.8
dg_60.genes	0.543	3.247	2.275	-0.814
dg_70.genes	0.758	3.519	2.36	-0.808
dg_80.genes	-0.51	1.49	0.274	-0.702

StDevs
FN_0.2	FN_0.4	FN_0.6	FN_0.8
dg_60.genes	715.6	356.157	102.149	16.79
dg_70.genes	706.966	345.468	102.763	15.79
dg_80.genes	561.116	283.134	84.097	13.27
```
